# Supplementary material for: Mesoscale functional organization and connectivity of color, disparity, and naturalistic texture in human second visual area
Source: eLife. 2025 Mar 20;13:RP93171. doi: 10.7554/eLife.93171 (PMC11925451; doi:10.7554/eLife.93171)
Supplement: Figure 2—figure supplement 1—source data 1. [file elife-93171-fig2-figsupp1-data1.docx]

Figure 2—figure supplement 1—source data 1. The number of stripes in manually defined ROIs

| Subjects | Thin stripe | Thick stripe | Pale stripe |
| --- | --- | --- | --- |
| S01 | 15 | 17 | 23 |
| S02 | 21 | 20 | 34 |
| S03 | 20 | 21 | 34 |
| S04 | 16 | 16 | 23 |
| S05 | 19 | 22 | 33 |
| S06 | 18 | 17 | 28 |
| S07 | 20 | 18 | 27 |
| S08 | 17 | 17 | 29 |
| S09 | 27 | 20 | 40 |
| S10 | 15 | 15 | 23 |
